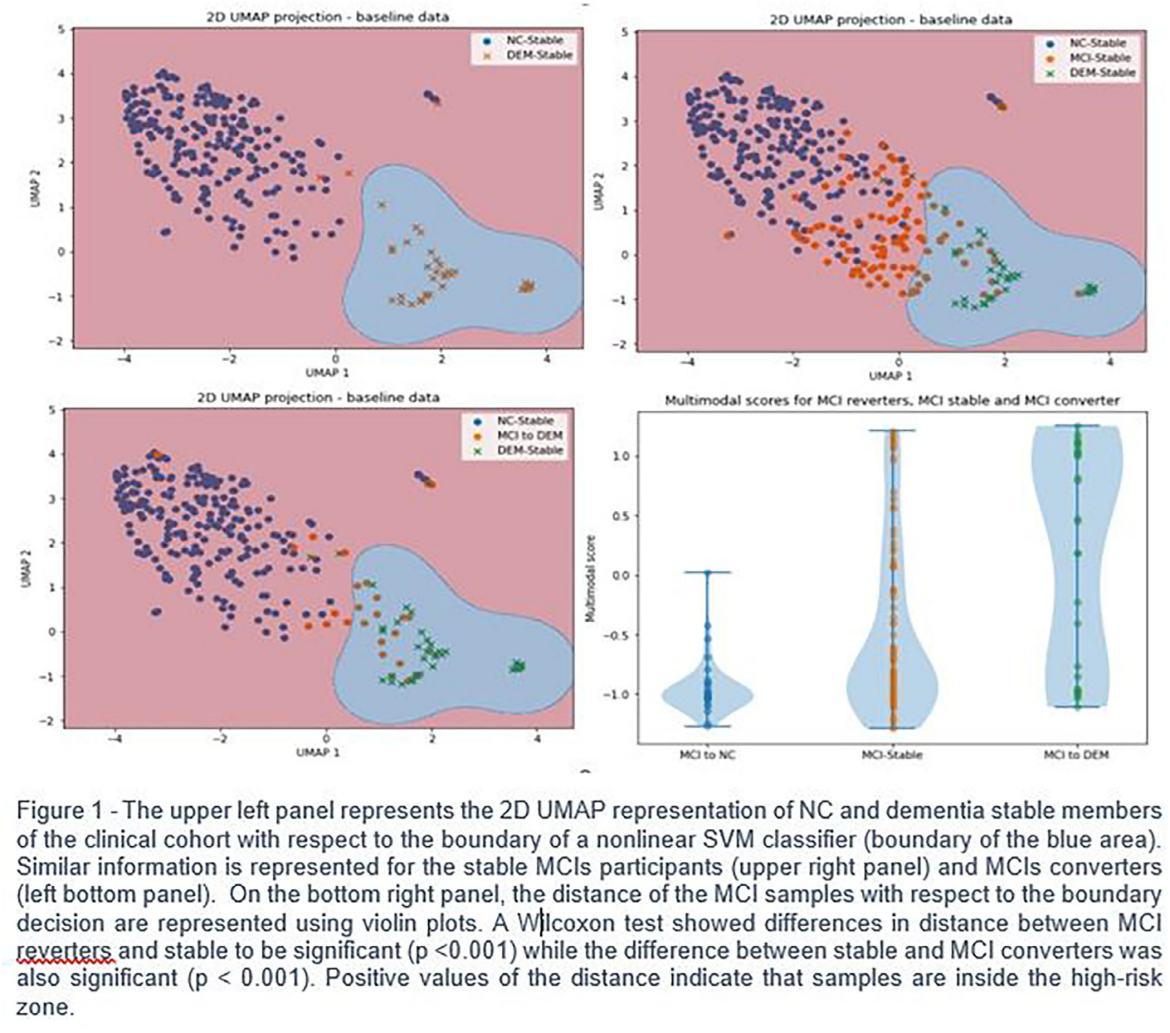# Characterizing dementia risk in individuals with mild cognitive impairment using manifold learning, blood biomarkers, cognitive and imaging data

**DOI:** 10.1002/alz.088014

**Published:** 2025-01-09

**Authors:** Ramon Casanova, Sarah A. Gaussoin, Lingyi Lu, Ryan Barnard, Tugce Duran, James R. Bateman, Samuel N. Lockhart, Gagan Deep, Timothy M. Hughes, Kathleen M. Hayden, Mark A. Espeland, Suzanne Craft

**Affiliations:** ^1^ Wake Forest University School of Medicine, Winston‐Salem, NC USA; ^2^ Wake Forest University School of Medicine, Winston Salem, NC USA

## Abstract

**Background:**

Uniform manifold approximation and projection (UMAP) is a technique for dimension reduction and visualization of high‐dimensional (HD) data. Here, we apply UMAP to represent in two dimensions, data from members of the Wake Forest School of Medicine Alzheimer’s Disease Research Center (WFUSM‐ADRC) clinical cohort.

**Methods:**

We examined baseline data from 542 WFUSM‐ADRC participants with mean age 70.1 years (range 54‐95 years), including 66% women and 18% Black self‐reported participants. A total of 195 participants were adjudicated with mild cognitive impairment(MCI), 67 with dementia and 280 were normal controls(NC) at baseline. Participants were further classified according to the stability of their cognitive status during 6.7 years of follow‐up with respect to baseline. We created a vector of 36 variables including cognitive tests, blood‐based biomarkers, MRI measures and age for each participant using baseline data. Based on UMAP, a 2D view of the data was created. Next, we fitted a nonlinear support vector machine with a Gaussian‐kernel and generated the boundary decision using data from participants classified at baseline as NC and dementia who kept the same status during their follow‐up. Data from individuals with MCI who remained stable, converted to dementia, or reverted to NC was provided to the classifier. The distance of each sample to the boundary was estimated. We evaluated this distance as a multimodal measure of AD dementia risk using a Wilcoxon test.

**Results:**

The classifier discriminated NC from dementia cases with an area under the curve of 96.3. The blue area in Figure 1 surrounded by the classifier’s boundary decision, we refer to as the “high‐risk zone”. Overall individuals with MCI falling inside the risk zone at baseline had significantly worse values of cognitive parameters (Verbal Fluency, AVLT, Digit Span, MoCA, Trails A &B), blood (GFAP, ptau181, NFL) and imaging biomarkers (AD Pattern Similarity scores, hippocampal and a temporal‐metaROI volume),(p < 0.001) than those individuals with MCI outside the high‐risk zone. Positive distances indicate the samples to be inside the zone. Differences in distances between the three MCIs groups were significant (p < 0.001).

**Conclusions:**

UMAP 2D projections have explanatory value to evaluate dementia risk in MCI.